# Test characteristics of thoracic point of care ultrasonography for the diagnosis of acute congestive heart failure in the emergency department

**DOI:** 10.1186/2036-7902-4-S1-A25

**Published:** 2012-12-18

**Authors:** Muhammad Tashkandi, Michael Woo, Christian Vaillancourt

**Affiliations:** 1 Department of Emergency Medicine, University of Ottawa, Ottawa, Ontario, Canada

## Introduction

The test characteristics of thoracic Point of Care Ultrasonography (PoCUS) for the diagnosis of acute congestive heart failure (CHF) are not well known, and no prior study evaluated the diagnostic impact of pleural effusions. We sought to determine the test characteristics of thoracic PoCUS when performed within 2 hours of initial emergency physician (EP) assessment by combining sonographic B-lines and pleural effusion to diagnose acute CHF.

## Methods

This prospective cohort study used a convenience sample of adult patients presenting to the ED with suspected acute CHF. An EP not involved in the patients care performed an eight-zone thoracic PoCUS (Positive when B-lines seen in >= 2 zones on each side). Two EPs blinded to thoracic PoCUS results performed a health record review that served as the criterion standard for CHF. We calculated the test characteristics of two (inferior lateral zones only) and eight-zone thoracic PoCUS with and without sonographic pleural effusion using stratified analysis with 95% confidence intervals (CI).

## Results

Of the 40 patients enrolled, 3 did not meet all inclusion criteria and were excluded. The mean age was 81, males 51%, 84% diagnosed with CHF, 54% arrived via EMS, 65% admitted, and 3% intubated. Positive and negative likelihood ratios, Sensitivity, and Specificity for the 37 patients analyzed are: 1) Eight-zone PoCUS: infinity; 0.35 (95% CI 0.22-0.57); 64.5% (95% CI 45%-80%); 100% (95% CI 51%-100%); 2) Eight-zone PoCUS with pleural effusion: infinity; 0.26 (95% CI 0.14-0.47); 74% (95% CI 55%-87%); 100% (95% CI 51-100%); 3) Twozone PoCUS: 4.43 (95% CI 0.7-27.7): 0.43 (95% CI 0.26-0.71): 63% (95%CI 44%-79%): 86% (95% CI 42%-99%); 4) Twozone PoCUS with pleural effusion: 5.4 (95% CI 0.9-32.5); 0.11 (95% CI 0.03-0.36); 90% (95% CI 73%-97%); 83% (95% CI 36%-99%).

## Conclusion

The test characteristics of thoracic PoCUS are improved with the inclusion of sonographic pleural effusion. There is a role for thoracic PoCUS in the diagnosis of acute CHF.

